# Trans-Cinnamaldehyde, Carvacrol, and Eugenol Reduce *Campylobacter jejuni* Colonization Factors and Expression of Virulence Genes *in Vitro*

**DOI:** 10.3389/fmicb.2017.00713

**Published:** 2017-04-25

**Authors:** Abhinav Upadhyay, Komala Arsi, Basanta R. Wagle, Indu Upadhyaya, Sandip Shrestha, Ann M. Donoghue, Dan J. Donoghue

**Affiliations:** ^1^Department of Poultry Science, University of ArkansasFayetteville, AR, USA; ^2^Poultry Production and Product Safety Research Unit, Agricultural Research Service (USDA)Fayetteville, AR, USA

**Keywords:** *Campylobacter jejuni*, virulence, attachment, invasion, CDT toxin, phytochemicals, gene expression

## Abstract

*Campylobacter jejuni* is a major foodborne pathogen that causes severe gastroenteritis in humans characterized by fever, diarrhea, and abdominal cramps. In the human gut, *Campylobacter* adheres and invades the intestinal epithelium followed by cytolethal distending toxin mediated cell death, and enteritis. Reducing the attachment and invasion of *Campylobacter* to intestinal epithelium and expression of its virulence factors such as motility and cytolethal distending toxin (CDT) production could potentially reduce infection in humans. This study investigated the efficacy of sub-inhibitory concentrations (SICs, concentration not inhibiting bacterial growth) of three GRAS (generally recognized as safe) status phytochemicals namely trans-cinnamaldehyde (TC; 0.005, 0.01%), carvacrol (CR; 0.001, 0.002%), and eugenol (EG; 0.005, 0.01%) in reducing the attachment, invasion, and translocation of *C. jejuni* on human intestinal epithelial cells (Caco-2). Additionally, the effect of these phytochemicals on *Campylobacter* motility and CDT production was studied using standard bioassays and gene expression analysis. All experiments had duplicate samples and were replicated three times on three strains (wild type S-8, NCTC 11168, 81–176) of *C. jejuni*. Data were analyzed using ANOVA with GraphPad ver. 6. Differences between the means were considered significantly different at *P* < 0.05. The majority of phytochemical treatments reduced *C. jejuni* adhesion, invasion, and translocation of Caco-2 cells (*P* < 0.05). In addition, the phytochemicals reduced pathogen motility and production of CDT in S-8 and NCTC 11168 (*P* < 0.05). Real-time quantitative PCR revealed that phytochemicals reduced the transcription of select *C. jejuni* genes critical for infection in humans (*P* < 0.05). Results suggest that TC, CR, and EG could potentially be used to control *C. jejuni* infection in humans.

## Introduction

The foodborne pathogen *Campylobacter* is the leading cause of bacterial gastroenteritis in humans resulting in an estimated 96 million annual infections globally (Kirk et al., 2015). In the United States, an estimated 1.3 million cases of Campylobacteriosis occur each year largely due to consumption of contaminated poultry products (Newell et al., 2010; CDC, 2014). Chickens act as the reservoir host of *Campylobacter*, wherein the pathogen colonizes the intestine and can persist for the entire lifespan of the birds without causing any disease. This leads to contamination of carcass during slaughter and increases the risk of human foodborne infections (Allen et al., 2007). After gaining entry through contaminated food in humans, *C. jejuni* attaches and invades the epithelial layer of lower intestinal tract (ileum, jejunum, colon) followed by epithelial cytopathy and enteritis (Dasti et al., 2010). In most cases, the infection consists of fever, headache, abdominal pain, vomiting, and diarrhea. However, in a minority of individuals, Campylobacteriosis triggers more serious illnesses such as Guillian-Barre Syndrome and Miller-Fisher syndrome that could lead to inflammatory polyneuropathy and fatal paralysis (EFSA, 2011; Silva et al., 2011). A plethora of virulence factors critical for attachment and invasion of epithelial cells, subsequent cytoplasmic proliferation, and cytopathy have been characterized for *C. jejuni* (Bolton, 2015). Major factors include motility systems (Young et al., 2007), attachment and invasion proteins (CadF, JlpA), and CDT production that causes cellular distension and cell death leading to enteritis (Silva et al., 2011). Thus, reducing the attachment and invasion of *C. jejuni* on intestinal epithelial cells and production of virulence factors such as motility and CDT could potentially control Campylobacteriosis in humans. Antibiotics such as macrolides (erythromycin, clarithromycin), and fluoroquinolones (ciprofloxacin, levofloxacin, moxifloxacin) are commonly used for treating *Campylobacter* infections in humans (Blaser and Engberg, 2008); however, there have been reports of development of resistance to these drugs (Engberg et al., 2001; Payot et al., 2006; Luangtongkum et al., 2009; Cha et al., 2016; Olkkola et al., 2016) and several resistance genes have been discovered in *Campylobacter* spp. (Gibreel et al., 2005; Gibreel and Taylor, 2006; Olkkola et al., 2016). This increase in antibiotic resistance along with reports of adverse drug reactions in patients (Periti et al., 1993; Thong and Tan, 2011) has fueled research exploring the potential of various antibiotic alternatives to combat *Campylobacter* infections in humans.

Since ancient times, plant extracts have been widely used as food preservatives, flavor enhancers, and dietary supplements for preventing food spoilage and improving human health. In addition, plant extracts are used in herbal medicine for treating various diseases. The antibacterial activity of several phytochemicals has been documented (Burt, 2004; Holley and Patel, 2005). A majority of these compounds are secondary metabolites produced during interaction between plants, animals, and microbes (Reichling, 2010). Trans-cinnamaldehyde (TC) is an aldehyde obtained from bark of cinnamon tree (*Cinnamomum zeylandicum*). Carvacrol (CR) or cymophenol is the principal antimicrobial ingredient in oregano oil (*Origanum vulgare*). Eugenol (EG) is yet another polyphenol compound that is the major antimicrobial component present in the oil of cloves (*Syzgium aromaticum*). All the aforementioned phytochemicals are classified as GRAS (Generally Recognized as Safe) by the FDA, and are approved for addition in food products (Food and Drug Administration, 2012, 2013).

Considerable literature exists on the antibacterial properties of phytochemicals that target cellular viability of bacteria; however, limited information is available on their effect in modulating the various aspects of bacterial virulence critical for causing disease in humans. The present study investigated the efficacy of sub-inhibitory concentrations (SICs, concentrations of compounds not inhibitory to bacterial growth) of TC, CR, and EG in reducing *C. jejuni* attachment, invasion, and translocation of human intestinal epithelial cells (Caco-2), and production of virulence factors *in vitro*. In addition, the effect of phytochemicals on the expression of critical virulence genes was investigated using real-time quantitative PCR.

## Materials and methods

### *Campylobacter* strains and culture conditions

All culture media were purchased from Difco (Becton Dickinson, Sparks, MD). Three strains of *C. jejuni*, including wild type S-8 (isolated from commercial broilers raised at University of Arkansas), NCTC 11168, and 81–176 (ATCC BAA-2151) were used in the study. Each strain was cultured separately in 10 ml of sterile Campylobacter Enrichment broth (CEB, Neogen, Lansing, MI) and incubated at 37°C for 48 h in a microaerophilic atmosphere (5% O_2_, 10% CO_2_, and 85% N_2_).

### Phytochemicals and determination of sub-inhibitory concentration (SIC)

The SIC of each phytochemical was determined using a previously published protocol (Amalaradjou et al., 2011) with slight modifications. Sterile 96-well polystyrene plates (Costar, Corning Incorporated, Corning, NY) containing serial dilutions of phytochemicals (Sigma-Aldrich) in CEB (100 μl/well) were inoculated with ~5.0 log CFU of *C. jejuni* in equal volume of CEB, followed by incubation at 37°C for 24 h. Bacterial growth was determined by culturing on *Campylobacter* Line Agar (CLA) plates (Line, 2001). The highest concentration of phytochemicals that did not inhibit the growth of *C. jejuni* after 24 h of incubation was selected as its respective SIC for the study. Since 100% ethanol was used as a diluent to increase solubility of phytochemicals for the experiments, its effect (at 0.1% concentration) on the various virulence attributes was also studied.

### Bacterial motility assay

The effect of TC, CR, and EG on *C. jejuni* motility was determined as described previously (Niu and Gilbert, 2004) with modifications. Separate petri dishes containing 25 ml of motility test medium (0.4% agar) with the respective SICs of each phytochemical were prepared. A mid-log culture (8 h) of *C. jejuni* was centrifuged at 3,600 g for 15 min and washed two times with Butterfield's phosphate diluent (BPD). Five microliters of washed culture (~7 log CFU/ml) was stab inoculated at the center of the motility medium, incubated in microaerophilic environment at 37°C for 24 h, and the zone of motility (bacterial migration distance from the site of stab) was measured.

### Cell culture

Human intestinal epithelial cells (Caco-2, ATCC HTB-37) were cultured in 25-cm^2^ tissue culture flasks (Falcon, Becton and Dickinson Company, Franklin Lakes, NJ) with minimum essential medium (DMEM, Gibco, Invitrogen, Carlsbad, CA) containing 10% (v/v) fetal bovine serum (Invitrogen). The cells were incubated at 37°C in a 5% (v/v) CO_2_ atmosphere.

### Attachment and invasion assay

The effect of SIC of phytochemicals (TC, CR, EG) on *C. jejuni* adhesion to and invasion of human epithelial cells was determined as previously reported (Koo et al., 2012). Monolayers of Caco-2 cells were grown in 24-well tissue culture plates (Costar) at ~10^5^ cells per well and inoculated with a mid-log culture (8 h) of *C. jejuni* ~6 log CFU/well (multiplicity of infection-10:1) either alone (control) or in combination with SIC of phytochemicals. The inoculated monolayers were incubated at 37°C for 1.5 h in a microaerophilic environment. For the adhesion assay, the inoculated monolayers (after 1.5 h of incubation) were rinsed three times in BPD and lysed with 1 ml of 0.1% Triton X-100 (Invitrogen, Carlsbad, CA). The number of adherent *C. jejuni* was determined by serial dilution and plating on CLA plates. For the invasion assay, the Caco-2 monolayer after 1.5 h of incubation (post-inoculation) was rinsed three times in minimal media and incubated for another 2 h in cell culture media containing gentamicin (100 μg/ml) to kill extracellular bacteria. Subsequently, the monolayer was treated with 0.1% triton X-100 as described above. The number of *C. jejuni* that invaded the epithelial cells was enumerated by serially diluting and plating the cell lysate on CLA plates followed by incubation at 37°C for 48 h for bacterial enumeration.

### Epithelial translocation assay

Bacterial epithelial translocation assay was performed as described previously (Koo et al., 2012). Caco-2 cells were cultured on transwell filter inserts (5-μm pore filter, Corning) placed in sterile 24-well tissue culture plates for 10–12 days to form a monolayer. Two hundred microliters of mid-log culture (8 h) of *C. jejuni* (6 log CFU/ml) inoculum was added to the apical well of the insert and incubated at 37°C for 3 h in a microaerophilic environment. Culture media (600 μl) from the basal well was plated on CLA agar for enumerating the number of *C. jejuni* that crossed the epithelial barrier.

### Cell viability assay for cytolethal distending toxin quantification

CDT activity in the supernatant of *C. jejuni* culture grown either alone (control) or in the presence of SICs of phytochemicals was quantified by cell viability assay (AbuOun et al., 2005; Lin et al., 2011) with modifications. Briefly, 1 ml of the 48 h culture of *C. jejuni* (grown with or without phytochemicals) was centrifuged at 10,000 g and the supernatant was collected. Serial 2-fold dilutions (100, 50, 25, 12.5, 6.25, 3.125, 1.56%) of the supernatant were made by mixing 100 μl of supernatant with 100 μl of cell culture medium and were added to Caco-2 cells followed by incubation for 9 days at 37°C. Negative control (0% cytotoxicity, obtained by addition of 200 μl of cell culture medium), media control (obtained by addition of appropriate volumes of CEB), and positive control (100% cytotoxicity, obtained by exposing the Caco-2 cells to cell lysis reagent) were also included for comparison. After 9 days, the amount of LDH release (indicator of cell cytotoxicity) was quantified using CytoTox-ONE homogeneous membrane integrity kit according to the manufacturer's instruction (Promega Inc., Madison, WI). Fluorescence was recorded (excitation wavelength of 560 nm and emission wavelength of 590 nm) on a Synergy 2 plate reader (BioTek, Higland Park, Winooski, VT) and percentage cytotoxicity was calculated according to the formula [Percent cytotoxicity = 100 × (LDH_T_/LDH_C_) where LDH_T_ refer to the difference in fluorescence between the sample and negative control (background fluorescence from media], and LDH_*C*_ refers to the difference in fluorescence between the positive control and negative control respectively.

### Real-time quantitative PCR (RT-qPCR)

The effect of phytochemicals on the expression of *C. jejuni* virulence genes was investigated using real-time quantitative PCR, as described previously (Upadhyay et al., 2012). Each *C. jejuni* strain was cultured separately with the higher SIC of phytochemicals at 37°C in CEB to mid-log phase (8 h) and total RNA was extracted using RNeasy RNA isolation kit (Qiagen, Valencia, CA). Complementary DNA was synthesized using the Superscript Reverse transcriptase kit (Invitrogen). The cDNA synthesized was used as the template for RT-qPCR. The primers for each gene (Table 1) were designed from published GenBank *C. jejuni* sequence using NCBI Primer design software. The amplification specificity was tested using NCBI-Primer BLAST, melt curve analysis and *in silico* PCR amplification (Bikandi et al., 2004). The amplified product was detected using SYBR Green reagent. Relative gene expression was determined using the comparative critical threshold (Ct) method on a Quant Studio 3 Real Time PCR system. Data were normalized to the endogenous control (16S rRNA gene), and the level of candidate gene expression between control and phytochemical treated sample was analyzed.

**Table 1:** **List of primers used for real-time qPCR analysis**.

**Gene with accession number**	**Primer**	**Sequence (5′–3′)**
16S-rRNA (NC_002163.1)	Forward	5′-TGAGGGAGAGGCAGATGGAA-3′
Endogenous control (Product length 78)	Reverse	5′-TCGCCTTCGCAATGGGTATT-3′
*motA* (NC_002163.1)	Forward	5′-AGCGGGTATTTCAGGTGCTT-3′
Flagellar motor protein A (Product length 75 bp)	Reverse	5′-CCCCAAGGAGCAAAAAGTGC-3′
*motB* (NC_002163.1)	Forward	5′-AATGCCCAGAATGTCCAGCA-3′
Flagellar motor protein A (Product length 51 bp)	Reverse	5′-AGTCTGCATAAGGCACAGCC -3′
*fliA* (NC_002163.1)	Forward	5′-AGCTTTCACGCCGTTACGAT-3′
Flagellar sigma factor (Product length 56 bp)	Reverse	5′-TCTTGCAAAACCCCAGAAGT-3′
*cadF* (NC_002163.1)	Forward	5′-CGCGGGTGTAAAATTCCGTC-3′
*Campylobacter* adhesion to fibronectin (Product length 135 bp)	Reverse	5′-TCCTTTTTGCCACCAAAACCA-3′
*ciaB* (NC_002163.1)	Forward	5′-TCTCAGCTCAAGTCGTTCCA -3′
*Campylobacter* invasion antigen B (Product length 50 bp)	Reverse	5′-GCCCGCCTTAGAACTTACAA -3′
*jlpA* (NC_002163.1)	Forward	5′-AGCACACAGGGAATCGACAG -3′
Jejuni lipoprotein A (Product length 66 bp)	Reverse	5′-TAACGCTTCTGTGGCGTCTT -3′
*cdtA* (NC_002163.1)	Forward	5′-TTTTGAAAATCGCCCTGCGG-3′
Cytolethal distending toxin A (Product length 57 bp)	Reverse	5′-GCTCCGCTAGGGCCTAAAAT-3′
*cdtB* (NC_002163.1)	Forward	5′-CTAGCGCAACTCAAGCAAGC-3′
Cytolethal distending toxin B (Product length 98 bp)	Reverse	5′-AATCGCAGCTAAAAGCGGTG-3′
*cdtC* (NC_002163.1)	Forward	5′-TAGCCCCTTGCACCCTAGAT-3′
Cytolethal distending toxin C (Product length 80 bp)	Reverse	5′-AGCAGCTGTTAAAGGTGGGG-3′
*cetA* (NC_002163.1)	Forward	5′-CCTACCATGCTCTCCTGCAC -3′
*Campylobacter* energy taxis protein (Product length 78 bp)	Reverse	5′-CGCGATATAGCCGATCAAACC-3′
*cetB* (NC_002163.1)	Forward	5′-GCCTTGTTGCTGTTCTGCTC -3′
*Campylobacter* energy taxis protein (Product length 88 bp)	Reverse	5′-TTCCGTTCGTCGTATGCCAA -3′
*racS* (NC_002163.1)	Forward	5′-AGACAAGTTGCCGAAGTTGC -3′
Reduced ability to colonize system Sensor (Product length 79 bp)	Reverse	5′-AGGCGATCTTGCCTACTTCA -3′
*racR* (NC_002163.1)	Forward	5′-AGAGAACAGCTTGTAAGTCGCT-3′
Reduced ability to colonize system Response regulator (Product length 83 bp)	Reverse	5′-ACCCTTAAGCGACCGATGAT -3′

### Statistical analysis

A completely randomized designed was used for the study. The bacterial counts were logarithmically transformed before analysis to achieve homogeneity of variance (Byrd et al., 2001). All experiments had duplicate samples and were replicated three times on three strains (wild type S-8, NCTC 11168, 81–176) of *C. jejuni*. Data from independent trials were pooled and analyzed using ANOVA with Fisher LSD test for multiple comparisons on GraphPad Prism ver 6.0. Differences were considered significant with *P*-values < 0.05.

## Results

The two SICs of phytochemicals that did not reduce *C. jejuni* (S-8, NCTC 11168, 81–176) growth as compared to respective control were 0.005, 0.01% for TC and EG, and 0.001, 0.002% for CR (data not shown). The SICs of phytochemicals did not change the pH of the culture media (*P* > 0.05).

### Effect of phytochemicals on *C. jejuni* motility

**Figure 1:**
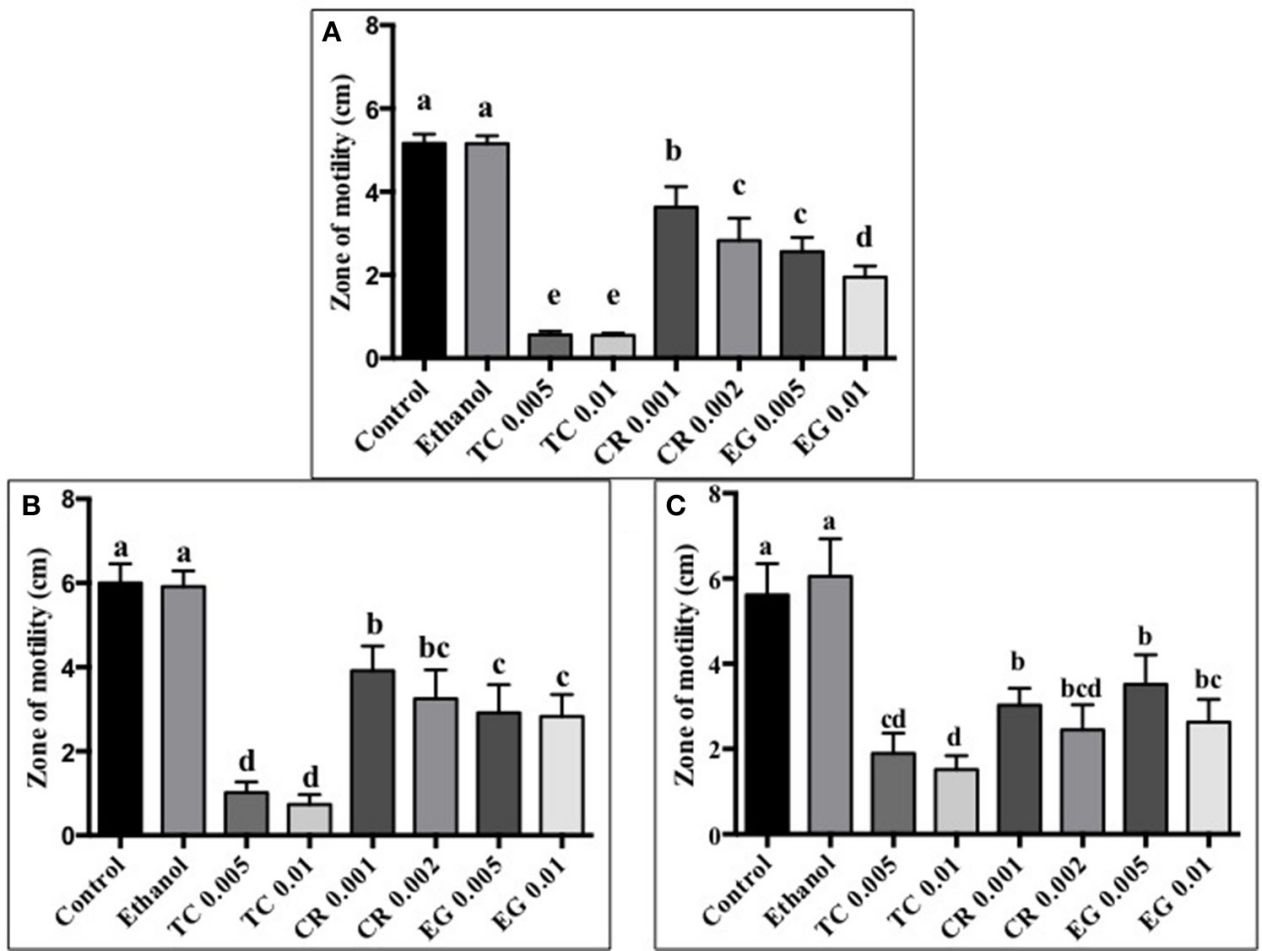
**Effect of phytochemicals on ***Campylobacter jejuni*** (A)** S-8 **(B)** NCTC 11168 **(C)** 81–176 motility. The treatments include control, ethanol, TC (0.005, 0.01%), CR (0.001, 0.002%), and EG (0.005, 0.01%). Error bars represent SEM (*n* = 6). Bars with different letters represent a significant difference between treatments (*P* < 0.05).

The effect of TC, CR, and EG on *C. jejuni* S-8 motility is presented in Figure 1A. All three phytochemicals at their respective SICs reduced *C. jejuni* S-8 motility (*P* < 0.05). TC was found to be the most effective treatment and decreased *C. jejuni* S-8 motility by >70% resulting in less than 1.0 cm zone as compared to control (5 cm) after 24 h incubation. Similar results were obtained with *C. jejuni* NCTC 11168 (Figure 1B) and *C. jejuni* 81–176 (Figure 1C) where all the phytochemical treatments significantly reduced pathogen motility.

### Effect of phytochemicals on *C. jejuni* attachment, invasion, and translocation of Caco-2 cells

**Figure 2:**
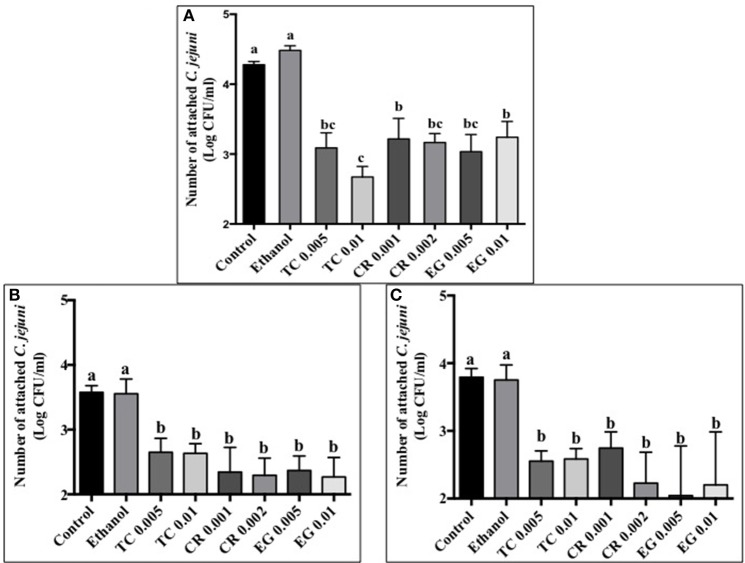
**Effect of phytochemicals on attachment of ***Campylobacter jejuni*** (A)** S-8 **(B)** NCTC 11168 **(C)** 81–176 to Caco-2 cells. The treatments include control, ethanol, TC (0.005, 0.01%), CR (0.001, 0.002%), and EG (0.005, 0.01%). Error bars represent SEM (*n* = 6). Bars with different letters represent a significant difference between treatments (*P* < 0.05).

**Figure 3:**
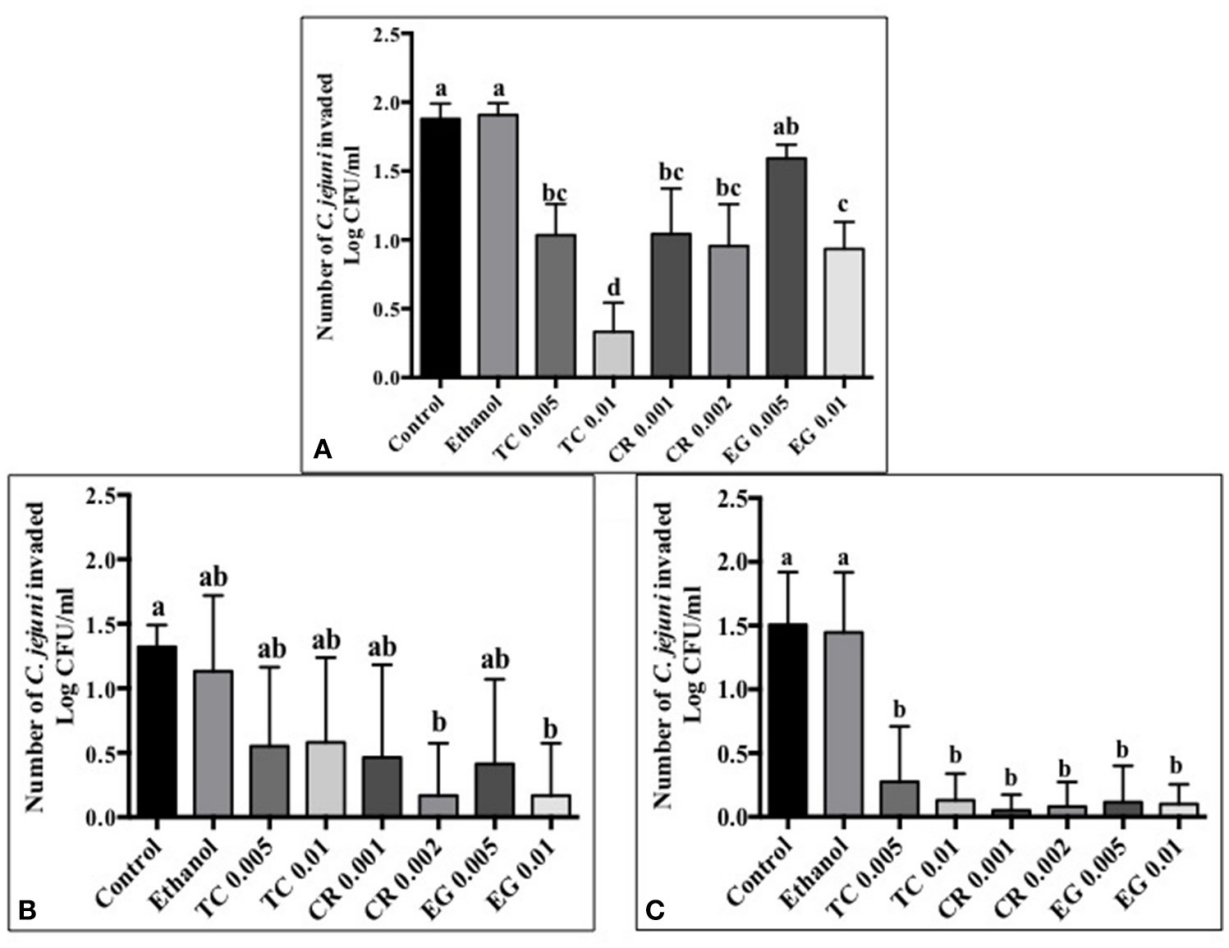
**Effect of phytochemicals on ***Campylobacter jejuni*** (A)** S-8 **(B)** NCTC 11168 **(C)** 81–176 invasion of Caco-2 cells. The treatments include control, ethanol, TC (0.005, 0.01%), CR (0.001, 0.002%), and EG (0.005, 0.01%). Error bars represent SEM (*n* = 6). Bars with different letters represent a significant difference between treatments (*P* < 0.05).

**Figure 4:**
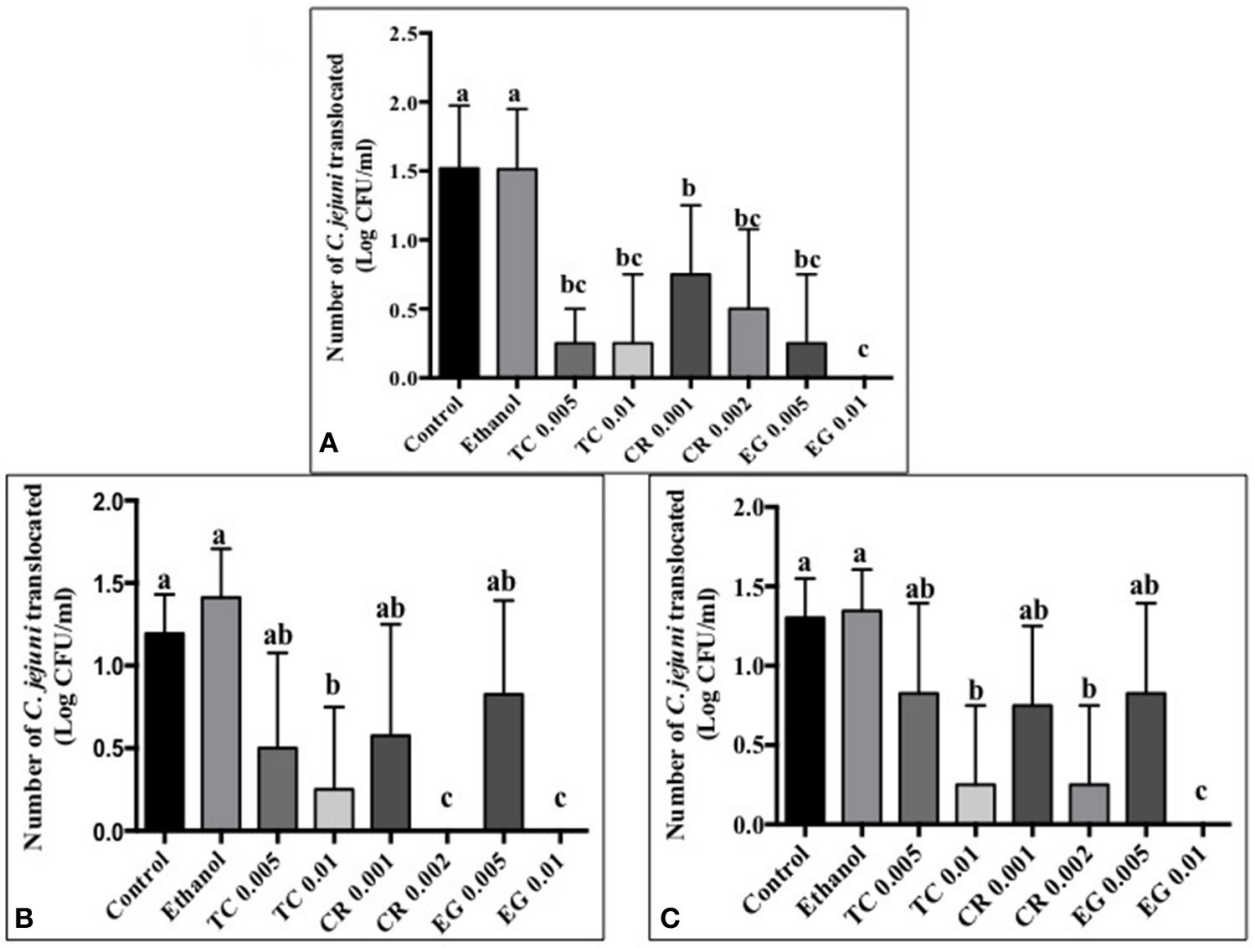
**Effect of phytochemicals on ***Campylobacter jejuni*** (A)** S-8 **(B)** NCTC 11168 **(C)** 81–176 translocation of Caco-2 cells. The treatments include control, ethanol, TC (0.005, 0.01%), CR (0.001, 0.002%), and EG (0.005, 0.01%). Error bars represent SEM (*n* = 6). Bars with different letters represent a significant difference between treatments (*P* < 0.05).

Tissue culture studies showed that the majority of phytochemical treatments decreased attachment, invasion and translocation of *C. jejuni* on Caco-2 cells in comparison to controls (Figures 2–4). For example, the SICs of TC, CR, and EG decreased attachment of *C. jejuni* S-8 by ~1–1.5 log CFU/ml as compared to control which had an attachment of ~4.2 log CFU/ml (Figure 2A). All phytochemicals except EG 0.005% reduced *C. jejuni* S-8 invasion as compared to controls (Figure 3A; *P* < 0.05). TC 0.01% was the most effective treatment and reduced the number of invading *C. jejuni* S-8 by ~1.5 log CFU/ml (*P* < 0.05). For TC and EG treatments, a concentration dependent reduction in *C. jejuni* S-8 invasion was observed. For translocation assay, all phytochemical treatments were able to significantly reduce *C. jejuni* S-8 translocation of Caco-2 cells (Figure 4A). The phytochemical treatments also reduced the attachment, invasion, and translocation of *C. jejuni* NCTC 11168 (Figures 2B,3B,4B) and *C. jejuni* 81–176 (Figures 2C,3C,4C). In case of *C. jejuni* NCTC 11168, a reduction of ~0.5–1 log CFU/ml was observed in adhesion to Caco-2 cells when exposed to phytochemicals (Figure 2B). Similarly, all phytochemical treatments reduced the attachment of *C. jejuni* 81–176 by at least 1 log CFU/ml as compared to control (Figure 2C). In case of invasion assay, both TC treatments, and lower SIC of CR and EG were not effective in reducing *C. jejuni* NCTC 11168 invasion (*P* > 0.05). However, the higher SIC of CR and EG reduced the invasion of all three strains of *C. jejuni* as compared to controls (*P* < 0.05). EG 0.01% treatment also exerted significant anti-translocation efficacy and completely inhibited the transfer of all three *C. jejuni* strains across the epithelial barrier (Figure 4; *P* < 0.05). None of the phytochemical treatments affected the health or integrity of Caco-2 cells (*P* > 0.05; data not shown).

### Effect of phytochemicals on *C. jejuni* induced cytotoxicity of Caco-2 cells

**Figure 5:**
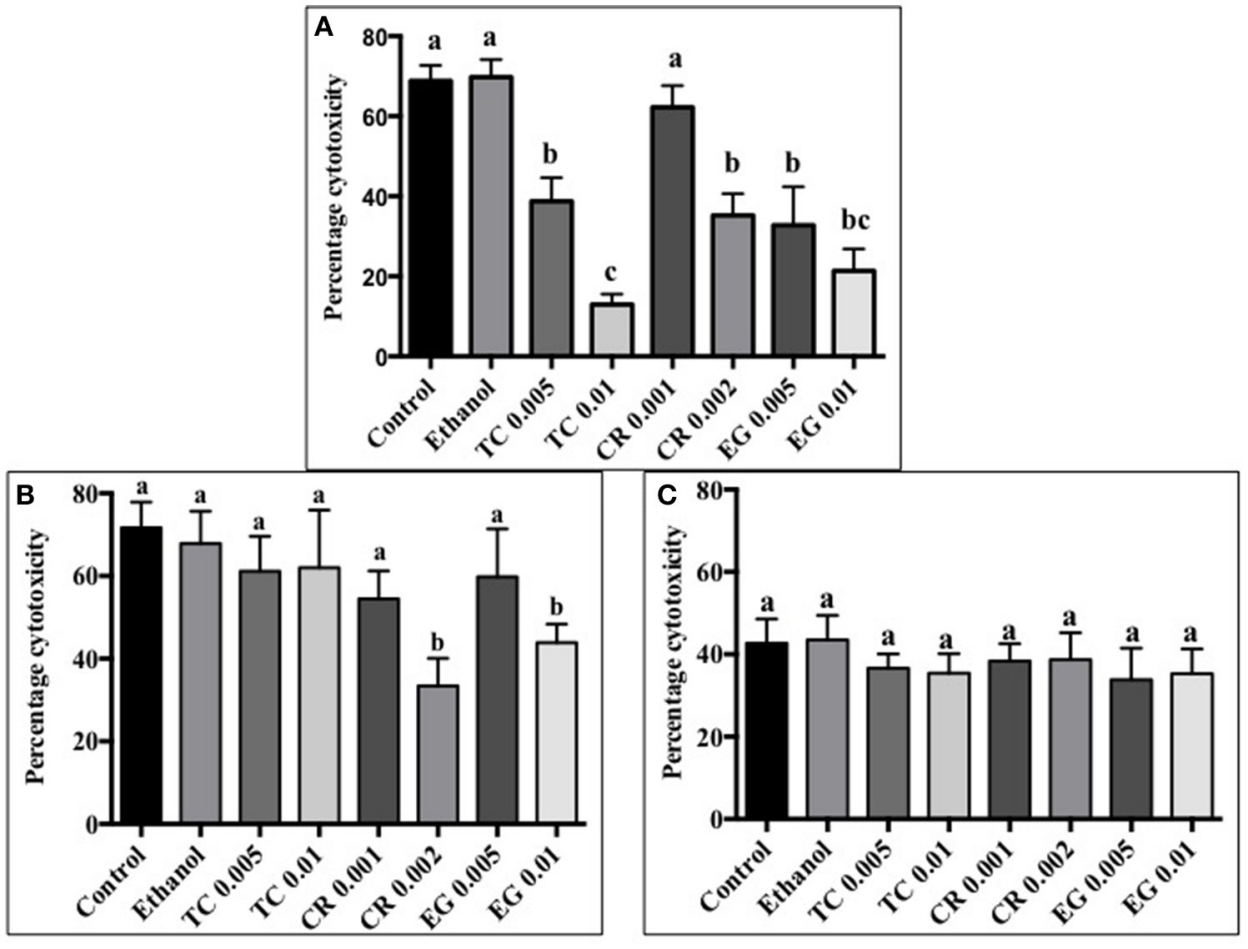
**Effect of phytochemicals on ***Campylobacter jejuni*** (A)** S-8 **(B)** NCTC 11168 **(C)** 81–176 induced cytotoxicity on Caco-2 cells. The treatments include control, ethanol, TC (0.005, 0.01%), CR (0.001, 0.002%), and EG (0.005, 0.01%). Error bars represent SEM (*n* = 6). Bars with different letters represent a significant difference between treatments (*P* < 0.05).

All phytochemicals except CR 0.001% reduced *C. jejuni* S-8 toxin induced cytotoxicity by at least 30% as compared to control (Figure 5A). A concentration dependent reduction in cell cytotoxicity was observed in TC treatments. The lower concentration of TC (0.005%) reduced cell cytotoxicity by ~30%, whereas the higher concentration (0.01%) reduced cell cytotoxicity by ~60% as compared to controls (*P* < 0.05). In case of *C. jejuni* NCTC 11168, only CR 0.002% and EG 0.01% were effective in reducing cell cytotoxicity by ~30% (Figure 5B; *P* < 0.05). None of the phytochemical treatments were able to reduce cell cytotoxicity mediated by *C. jejuni* 81–176 (Figure 5C; *P* > 0.05).

### Effect of phytochemicals on expression of *C. jejuni* virulence genes

Effect of phytochemicals on expression of virulence genes in the three strains of *C. jejuni* is presented in Table 2. Real-time quantitative PCR results revealed that TC 0.01% down-regulated the expression of virulence genes coding for motility (*fliA*), attachment (*cadF*) and toxin production (*cdtA, cdtB, cdtC*) in *C. jejuni* S-8. CR and EG did not significantly reduce the expression of the tested virulence genes in *C. jejuni* S-8 except for *racR* by EG. Interestingly, many of the virulence genes were upregulated by CR and EG treatments (*P* < 0.05). The ethanol treatments did not affect the expression of the tested virulence genes in *C. jejuni* S-8 (*P* > 0.05). In case of *C. jejuni* NCTC 11168, CR 0.002%, and EG 0.01% treatments reduced the expression of *motA, cadF, cdtB*, and *motA, cdtA* genes respectively (*P* < 0.05). The expression of the tested genes was not affected by ethanol treatment (*P* > 0.05). None of the phytochemical treatments were able to significantly reduce the expression of tested genes in *C. jejuni* 81–176 (*P* > 0.05). Interestingly, the expression of select genes in *C. jejuni* NCTC 11168 (*motB, jlpA*) and *C. jejuni* 81–176 (*motA, motB, cadF, jlpA, cdtA,B,C*) was upregulated by 0.01% TC treatment and CR 0.002, EG 0.01% treatments respectively (*P* < 0.05).

**Table 2:** **Effect of TC 0.01%, CR 0.002%, EG 0.01%, and ethanol on the expression of ***Campylobacter jejuni*** (S-8, NCTC 11168, 71–176) virulence genes**.

**Gene**	**Relative fold change (Log**_**10**_**RQ)**					
	**TC 0.01%**			**CR 0.002%**		
	**S-8**	**11168**	**81–176**	**S-8**	**11168**	**81–176**
*motA*	−0.2 ± 0.2^a^	0.05 ± 0.4^ab^	0.6 ± 0.2^b^	−0.2 ± 0.07^a^	−0.7 ± 0.1^b^^*^	1.5 ± 0.09^c^^*^
*motB*	−0.1 ± 0.1^a^	0.5 ± 0.02^a^^*^	0.5 ± 0.7^a^	−0.009 ± 0.03^a^	−0.2 ± 0.3^a^	1.7 ± 0.05^b^^*^
*fliA*	−0.3 ± 0.2^a^^*^	0.3 ± 0.08^a^	0.1 ± 0.1^a^	−0.06 ± 0.1^a^	0.08 ± 0.2^a^	0.08 ± 0.1^a^
*cadF*	−0.3 ± 0.2^a^^*^	−0.09 ± 0.1^a^	0.2 ± 0.6^a^	−0.2 ± 0.1^a^	−0.6 ± 0.2^a^^*^	1.2 ± 0.03^b^^*^
*ciaB*	0.07 ± 0.04^a^	0.2 ± 0.06^a^	0.3 ± 0.07^a^	−0.1 ± 0.06^a^	0.1 ± 0.2^a^	0.2 ± 0.2^a^
*jlpA*	−0.09 ± 0.08^a^	0.6 ± 0.1^a^^*^	0.6 ± 0.3^a^	−0.06 ± 0.04^a^	−0.1 ± 0.1^a^^*^	1.7 ± 0.2^b^^*^
*cdtA*	−0.4 ± 0.1^a^^*^	0.3 ± 0.07^ab^	0.5 ± 0.3^b^	−0.1 ± 0.06^a^	−0.3 ± 0.1^a^	1.7 ± 0.06^b^^*^
*cdtB*	−0.5 ± 0.19^a^^*^	0.08 ± 0.1^ab^	0.6 ± 0.2^b^	−0.2 ± 0.04^a^	−0.4 ± 0.1^a^^*^	1.9 ± 0.1^b^^*^
*cdtC*	−0.4 ± 0.17^a^^*^	0.0004 ± 0.2^ab^	0.5 ± 0.3^b^	−0.09 ± 0.1^a^	−0.1 ± 0.1^a^	1.8 ± 0.2^b^^*^
*cetA*	−0.05 ± 0.04^a^	0.1 ± 0.1^a^	0.2 ± 0.1^a^	0.3 ± 0.11^a^^*^	0.05 ± 0.05^a^	0.2 ± 0.2^a^
*cetB*	−0.08 ± 0.04^a^	0.3 ± 0.07^a^	0.06 ± 0.1^a^	0.2 ± 0.007^a^	0.29 ± 0.07^a^	0.3 ± 0.07^a^
*racS*	−0.2 ± 0.07^a^	0.19 ± 0.06^a^	0.1 ± 0.1^a^	0.4 ± 0.04^a^^*^	0.3 ± 0.07^a^	0.3 ± 0.1^a^
*racR*	−0.1 ± 0.07^a^	0.18 ± 0.1^a^	0.1 ± 0.2^a^	0.2 ± 0.05^a^	0.03 ± 0.1^a^	0.2 ± 0.03^a^
**Gene**	**EG 0.01%**			**Ethanol**		
*motA*	0.2 ± 0.09^a^	−0.7 ± 0.06^b^^*^	1.2 ± 0.1^c^^*^	0.04 ± 0.05^a^	0.03 ± 0.1^a^	0.06 ± 0.1^a^
*motB*	0.4 ± 0.07^a^^*^	−0.4 ± 0.2^b^	1.4 ± 0.2^c^^*^	−0.02 ± 0.2^a^	0.3 ± 0.1^a^	0.1 ± 0.1^a^
*fliA*	0.2 ± 0.07^a^	0.08 ± 0.05^a^	0.2 ± 0.1^a^	−0.03 ± 0.1^a^	0.4 ± 0.08^b^	0.08 ± 0.1^ab^
*cadF*	0.3 ± 0.1^a^^*^	0.12 ± 0.1^a^	0.9 ± 0.1^b^^*^	0.08 ± 0.06^a^	−0.1 ± 0.01^a^	−0.1 ± 0.3^a^
*ciaB*	0.2 ± 0.08^a^	0.4 ± 0.1^a^	0.5 ± 0.2^a^	0.03 ± 0.04^a^	0.3 ± 0.01^a^	0.1 ± 0.09^a^
*jlpA*	0.3 ± 0.1^a^^*^	−0.2 ± 0.2^a^	1.4 ± 0.1^a^^*^	0.03 ± 0.04^a^	0.15 ± 0.1^a^	−0.04 ± 0.1^a^
*cdtA*	0.3 ± 0.08^a^^*^	−0.4 ± 0.08^b^^*^	1.3 ± 0.1^c^^*^	0.1 ± 0.03^a^	0.16 ± 0.08^a^	−0.04 ± 0.1^a^
*cdtB*	0.03 ± 0.07^a^	0.01 ± 0.09^a^	1.4 ± 0.1^b^^*^	0.06 ± 0.02^a^	0.1 ± 0.1^a^	0.1 ± 0.1^a^
*cdtC*	0.1 ± 0.1^a^	0.1 ± 0.1^a^	1.08 ± 0.1^b^^*^	−0.17 ± 0.1^a^	−0.1 ± 0.1^a^	0.03 ± 0.1^a^
*cetA*	0.3 ± 0.03^a^^*^	0.08 ± 0.06^a^	0.2 ± 0.07^a^	−0.02 ± 0.2^a^	0.09 ± 0.08^a^	0.1 ± 0.04^a^
*cetB*	0.3 ± 0.1^a^^*^	0.11 ± 0.1^a^	0.4 ± 0.05^a^	0.08 ± 0.06^a^	−0.03 ± 0.09^a^	−0.1 ± 0.1^a^
*racS*	−0.1 ± 0.14^a^	0.32 ± 0.1^b^	−0.1 ± 0.1^a^	0.16 ± 0.03^a^	−0.01 ± 0.1^a^	0.3 ± 0.1^a^
*racR*	−0.3 ± 0.01^a^^*^	0.06 ± 0.04^b^	0.2 ± 0.02^b^	0.03 ± 0.04^a^	−0.3 ± 0.2^a^	−0.07 ± 0.1^a^

*Values represent Mean ± SEM. Control had a basal RQ of 1 (log_10_ RQ = 0)*.

* *Relative fold change significantly different from control. Superscripts with different letters in a row represent significant difference between C. jejuni strains for a treatment*.

## Discussion

The colonization of enteric pathogens in the gut and their interaction with the human intestinal cells is central to most infections and illnesses. The poultry associated foodborne pathogen *C. jejuni* expresses an array of virulence factors that orchestrate its pathophysiology in humans. In this study we investigated the effect of phytochemicals trans-cinnamaldehyde, carvacrol, and eugenol in reducing the expression of virulence factors of *C. jejuni in vitro* as a first step before conducting future *in vivo* studies.

The motility imparted by the polar flagella of *C. jejuni* is essential for colonization of the mucus lining of the intestinal tract and subsequent attachment and invasion of intestinal epithelium (Guerry, 2007). Our results revealed that TC, CR, and EG significantly decreased *C. jejuni* motility (Figures 1A–C). In addition, majority of phytochemical treatments at their higher SIC also reduced attachment, invasion and translocation of *C. jejuni* through intestinal epithelial cells (Figures 2–4). (van Alphen et al., 2012) had similar findings and observed that pre-exposure of *C. jejuni* to low concentrations of carvacrol modulated motility and invasion of INT-407 intestinal epithelial cells without affecting intracellular ATP levels or epithelial function. Reduced motility and attachment efficiency in *C. jejuni* on exposure to clove oil (Kovács, 2014), thyme (Pogacar et al., 2016), herbal extracts (Bensch et al., 2011), and campynexins (Johnson et al., 2015) have also been observed.

After invading the human intestinal epithelium, *C. jejuni* produces the tripartite CDT toxin. The catalytic domain (CdtB) localizes in the nucleus leading to DNA damage, cell-cycle arrest, and cytotoxicity (Pickett et al., 1996; Lara-Tejero and Galán, 2000; Young et al., 2007). The activity of CDT was first described in culture supernatants that caused eukaryotic cells to slowly distend over a period of 2–5 days, eventually leading to cell death (Johnson and Lior, 1988). We observed that majority of phytochemical treatments significantly reduced the *C. jejuni* S-8 CDT mediated cell cytotoxicity in Caco-2 cells (Figure 5A). Previously, Castillo et al. (2011) demonstrated that extracts from *A. farnesiana* and *A. ludoviciana* inhibited cytotoxin production in *C. jejuni* and *C. coli*. In another study, Gillespie et al. (2013) screened 30,000 small molecules for toxin inhibition activity and observed that 4-bromobenzaldehyde N-(2, 6-dimethyphenyl) semicarbazone (EGA) inhibited intoxication by CDT derived from *Haemophilus ducreyi* and *Escherichia coli*. Although the phytochemical treatments were found to be effective against *C. jejuni* S-8 mediated cell cytotoxicity, considerable strain based variation in their efficacy was observed in our assay (Figures 5B,C). We believe that this result is due to variations in the genomic composition of the three strains tested that could potentially alter the molecular targets of phytochemicals. For example, Jeon et al. (2005) reported that a mutation in *luxS* gene (coding for quorum sensing) affected the transcription of CDT operon. Since phytochemicals are known to exert their effect by modulating *luxS* and quorum sensing (Koh and Tham, 2011; Nazzaro et al., 2013; Upadhyay et al., 2013), a disruption of their efficacy due to changes in target genes coding for quorum sensing is possible.

It has been previously shown that sub-inhibitory or sub-lethal concentrations of antimicrobials modulate the transcription of genes in various bacterial pathogens (Fonseca et al., 2004; Tsui et al., 2004; Upadhyay et al., 2012) including *C. jejuni* (Arambel et al., 2015; Oh and Jeon, 2015). Since the sub-inhibitory concentrations of phytochemicals did not inhibit the growth of *C. jejuni*, the reduction observed in *C. jejuni* virulence attributes could be due to the effect of phytochemicals on the transcription of virulence genes. Therefore, we used RT-qPCR to determine the effect of phytochemicals on the expression of genes, which have been previously reported to contribute to the *C. jejuni* infection process in humans. We selected an incubation time of 8 h since we wanted to investigate the effect of phytochemicals on the expression of virulence genes of *C. jejuni* over extended exposure time, as would be the case in the human gut. 16S rRNA gene was selected as endogenous control since it was stable in its expression between control and treatment groups. Several other researchers have also used 16S rRNA gene as endogenous control for studying the expression of virulence genes in pathogens (McKillip et al., 1998; Tasara and Stephan, 2007; Xue et al., 2008; Hays, 2009; Atshan et al., 2013; Schroeder et al., 2015) including *C. jejuni* (Koolman et al., 2016). The flagellar biosynthesis gene, *fliA* regulates several genes involved in *Campylobacter* motility. Genes *motA* and *motB* code for flagella motor function and aid in motility and colonization (Hermans et al., 2011). *cetA* and *cetB* are energy taxis genes that contribute to directional motility of *Campylobacter* in response to changes in external environment. The *Campylobacter* surface protein CadF in combination with CiaB (Young et al., 2007) and JlpA mediates binding of the pathogen to host epithelial cells through fibronectin-mediated attachment thereby facilitating colonization (Konkel et al., 1999, 2005; Monteville and Konkel, 2002; Monteville et al., 2003). RacS-RacR is another important two-component regulatory system that plays a role in temperature dependent growth and colonization in *C. jejuni* (Brás et al., 1999). In addition to the aforementioned attachment and motility factors, *C. jejuni* produces CDT encoded by *cdtA, cdtB*, and *cdtC* genes. The subunits CdtA and CdtC associate with the nuclease CdtB to form a tripartite complex that translocate CdtB into the host cell leading to arrest in cell cycle, cell death and enteritis (Young et al., 2007). We observed that phytochemicals modulated the expression of some of the virulence genes tested and this varied among the three strains of *C. jejuni*. For example, SIC of TC decreased the expression of genes coding for motility *(fliA)* and attachment (*cadF*) in strain S-8 (Table 2). In addition, TC down-regulated the transcription of *cdtA, cdtB*, and *cdtC* genes that contribute to CDT-mediated cell lysis in *C. jejuni* S-8. Thus, the reduced cell cytotoxicity observed in response to TC (Figure 5A) could potentially be due to the effect of TC on the expression of related genes in *C. jejuni* S-8. We did not observe significant down-regulation in the expression of *cdtA,B,C* genes in response to TC in *C. jejuni* NCTC 11168 and *C. jejuni* 81–176 (Table 2). This was reflected in the phenotypic assay as well where TC did not significantly reduce cell cytotoxicity in the two strains (Figures 5B,C). Similarly, CR down-regulated the expression of genes critical for motility (*motA*), attachment (*cadF*), and CDT production (*cdtB*) in *C. jejuni* NCTC 11168, whereas it did not reduce the expression of tested genes in wild type S-8 or 81–176 (Table 2). A directly related finding to this gene expression result was observed in the cell cytotoxicity assay where only higher SIC of CR reduced *C. jejuni* NCTC 11168 mediated cell cytoxicity (Figure 5B) whereas none of the CR treatments reduced *C. jejuni* 81–176 mediated cell cytotoxicity of Caco-2 cells (Figure 5C). As observed with TC and CR, EG also affected the expression of genes in the three strains differently. EG 0.01% treatment significantly reduced the expression of *racR* in *C. jejuni* S-8 and *motA, cdtA* genes in *C. jejuni* NCTC 11168 (Table 2), whereas the expression of genes in *C. jejuni* 81–176 was not reduced. These results indicate that phytochemicals exert their anti-virulence effect via only selected genes and this could vary among different strains of the pathogen. Previously, Lee et al. (2012) showed that flavones could exert anti-virulence effect by modulating only selected genes (*sae* and *agr*) in *Staphylococcus aureus*. Expression of genes such as *sigB* (RNA polymerase sigma factor) and *sar* (accessory regulator A) was not affected by flavones. In a recent study, Singh et al. (2016) reported that eugenol has strong binding affinity for surface exposed lysines in proteins. The essential oil inhibited formation of glycation end products by binding to ε-amine group on lysine. In another study, carvacrol was found to inhibit the binding of nicotine to nicotinic acetylcholine receptor (Tong et al., 2013) indicating that these phytochemicals could be exerting their anti-virulence properties through modulating receptor binding of virulence proteins in *C. jejuni* in addition to their effect on gene expression. Moreover, carvacrol has been shown to modulate the expression of HSP60 (GroEL) chaperones and other proteins that affect protein folding in *Escherichia coli* O157:H7 (Burt et al., 2007) and *Listeria monocytogenes* (Guevara et al., 2015). Therefore, it is possible that phytochemicals may be exerting their anti-virulence effect against *C. jejuni* via similar mechanism(s) that affect protein folding.

In conclusion, our study showed that phytochemicals TC, CR, and EG were effective in reducing the virulence attributes of *C. jejuni* critical for causing infection in humans. Since phytochemicals including TC, CR, and EG have been found to be stable in the gastrointestinal tract of monogastric animals (Si et al., 2006; Michiels et al., 2008), these plant compounds could potentially be used as dietary supplements to control *C. jejuni* infection in humans. However, *in vivo* studies in an appropriate mammalian model along with genomic characterization, transcriptomic and proteomic profiling of pathogens are necessary to validate the antimicrobial efficacy, mechanism of action, and safety of the plant compounds.

## Author contributions

AU designed the study. AU, KA, BW, IU, and SS conducted the experiments. AU analyzed the data and wrote the manuscript. AD and DD critically analyzed and revised the manuscript.

### Conflict of interest statement

The authors declare that the research was conducted in the absence of any commercial or financial relationships that could be construed as a potential conflict of interest.
